# Evaluation of segmental scleral buckling surgery for stage 4A retinopathy of prematurity in China

**DOI:** 10.3389/fmed.2022.969861

**Published:** 2022-08-03

**Authors:** Yusheng Zhong, Yating Yang, Hong Yin, Mingwei Zhao, Xiaoxin Li, Jianhong Liang, Yong Cheng

**Affiliations:** ^1^Department of Ophthalmology and Clinical Centre of Optometry, Peking University People’s Hospital, Eye Diseases and Optometry Institute, Beijing Key Laboratory of Diagnosis and Therapy of Retinal and Choroid Diseases, College of Optometry, Peking University Health Science Center, Beijing, China; ^2^Xiamen Eye Center, Xiamen University, Xiamen, China

**Keywords:** scleral buckling, retinopathy of prematurity, stage 4A, segmental, surgery

## Abstract

**Aims:**

To describe the long-term effect of scleral buckling (SB) surgery for stage 4A retinopathy of prematurity (ROP).

**Methods:**

A retrospective chart review was conducted for patients with a diagnosis of stage 4A ROP who underwent SB between October 2010 and October 2021. Basic data were collected from patient charts, including gender, birth weight, gestational age at birth, disease stage, presence of plus disease, preoperative treatment [laser photocoagulation, intravitreal anti-vascular endothelial growth factor (VEGF) agent therapy, or a combination of both] and complications (vitreous hemorrhages), postmenstrual age at surgery, intraoperative combined treatment, and total length of follow-up. Retinal attachment status after surgery, postoperative complications (glaucoma, cataract), date and type of subsequent retinal surgeries (if performed), and refractive status 1 year after surgery were evaluated. The follow-up time after the first procedure was over 1 year.

**Results:**

Six-two eyes from forty-eight patients met the inclusion criteria for this study. The initial reattachment rate was 93.5% (58/62 eyes), and the final reattachment rate was 100% after two procedures at the end of follow-up. The incidence of cataracts was 3.2% (2/62), with no eye subsequently needing lensectomy surgery. None of the patients developed glaucoma during the follow-up time. The average spherical equivalent refraction value for patients was −3.00 ± 2.51 D (−7.60 D to +2.75 D) 1 year after surgery.

**Conclusion:**

SB, especially segmental buckling, which induces less myopia and does not require buckle removal, has the potential to provide a significant positive impact in the treatment of stage 4A ROP.

## Introduction

Retinopathy of prematurity (ROP) is a leading cause of childhood blindness that occurs in premature infants, especially in the most immature infants throughout the world (1, 2). Although the advent of retinal ablation and intravitreal injection of anti-vascular endothelial growth factor (VEGF) drugs have improved the prognosis in threshold and prethreshold ROP, some treated eyes still experience retinal detachment (3, 4).

Surgical intervention in the early stage can yield the best chances of retinal reattachment and better functional outcomes for these eyes (5). Multiple studies have shown that scleral buckling (SB) and lens-sparing vitrectomy (LSV) are well-established techniques for the repair of ROP-related retinal detachment (6, 7). In previous literature, the success rate of LSV was higher than SB (8, 9). However, we believe that stage 4A ROP is partial retinal detachment, many of which occur in the periphery of the retina. LSV may damage the lens, resulting in cataract development and the need for cataract surgery. In addition, some studies (10, 11) have reported that LSV can result in potentially iatrogenic retinal breaks, vitreous hemorrhage and infection, which lead to a poor outcome.

It is universally acknowledged that SB can certainly neutralize the tractional forces of the extraretinal proliferation and effectively stabilize the neovascular activity of the fibrovascular tissue (12, 13). As an external surgery, SB is less invasive and carries less risk of surgical complications. However, few investigations have been published specifically focusing on the efficacy of SB for stage 4A ROP to date. Therefore, the aim of this study was to report on the long-term anatomic outcomes and complications after early SB for infants with stage 4A ROP.

## Materials and methods

This was a single-center, retrospective, consecutive case series of premature infants who underwent SB surgery for stage 4A ROP from January 2010 to October 2021. This study was approved by the Ethics Committee and Institutional Review Board of Peking University People’s Hospital. Written informed consent was obtained from the guardian of each participant in accordance with the Declaration of Helsinki. The stage of retinal detachment for each patient was determined during an examination under anesthesia according to the International Classification of Retinopathy of Prematurity (14). SB was selected as the most appropriate treatment for the included patient by experienced surgeons based on their best clinical judgment. We would prefer to perform SB on patients with stage 4A ROP whose tractional retinal detachment within six clock hours. All these treated cases were consecutive cases. Besides, if the patient was known any other eye disease except ROP was excluded.

The baseline characteristics of the patients, including gender, birth weight, gestational age at birth, disease stage, presence of plus disease, preoperative treatment (laser photocoagulation, intravitreal anti-VEGF agent therapy, or a combination of both) and complications (vitreous hemorrhage), postmenstrual age at surgery, intraoperative combined treatment, and total length of follow-up, were collected. Retinal attachment status after surgery, postoperative complications (glaucoma, cataract), date and type of subsequent retinal surgeries (if performed), and refractive status 1 year after surgery were evaluated.

The SB technique used in this series has been described in detail previously (15), and in this series, segmental SB was performed for all of the patients. All eyes were operated on by three surgeons (HY, JL, and YC) in the same group using the same surgical procedures and standards. The precise range and location of the tractional retinal detachment was marked on the scleral after a thorough examination under binocular indirect ophthalmoscopy. The silicone sponge was sutured to the marked area with 5-0 nylon suture. Subretinal fluid was not aspirated. Follow-up examinations were routinely performed in the first week after surgery and then at 1 month and every 3–6 months thereafter. At the follow-up sessions, all patients underwent a detailed ophthalmic examination under anesthesia, including ocular tonometry, cycloplegic refraction and a dilated fundus examination. Anatomical success was defined as total attachment of the retina. The refractive status was recorded as the spherical equivalent (SE, spherical power plus half of the cylindrical power), which was measured by a handheld autorefractometer (FR-5000; Grand Seiko Co., Ltd., Hiroshima, Japan). The average reading was obtained with at least three measurements. The follow-up time was at least 1 year after the first procedure.

The research data were uploaded to a computer environment and evaluated using SPSS V.26.0 (SPSS TM, IBM). Descriptive statistics are presented as the mean ± SD, median (minimum–maximum), frequency distribution and percentage.

## Results

Our cohort consisted of 62 eyes of 48 children, of whom 24 were male and 24 were female, with an average gestational age of 29.5 ± 1.71 weeks (range 26.6–33.0 weeks) and a birth weight of 1,335.8 ± 322.93 g (range 840–2,300 g). The mean postmenstrual age at surgery was 50.7 ± 9.59 weeks (range 38.7–93.3 weeks). The mean time of the follow-up was 3.64 ± 2.53 years (range 1–10.48 years). The baseline data of the patients are presented in Table 1.

**TABLE 1:** Baseline data of the patients who underwent scleral buckling surgery for stage 4A ROP.

	Stage 4A
Eyes, *n*	62
Patients, *n*	48
Gender, male, %	24 (50%)
Mean gestational age ± SD weeks (range)	29.5 ± 1.71 (26.6–33)
Mean birth weight ± SD gram (range)	1,335.8 ± 322.93 (840–2,300)
Mean postmenstrual age at surgery ± SD weeks (range)	50.7 ± 9.59 (38.7–93.3)
Mean follow-up duration ± SD years (range)	3.64 ± 2.53 (1–10.48)

SD, standard deviation.

**FIGURE 1:**
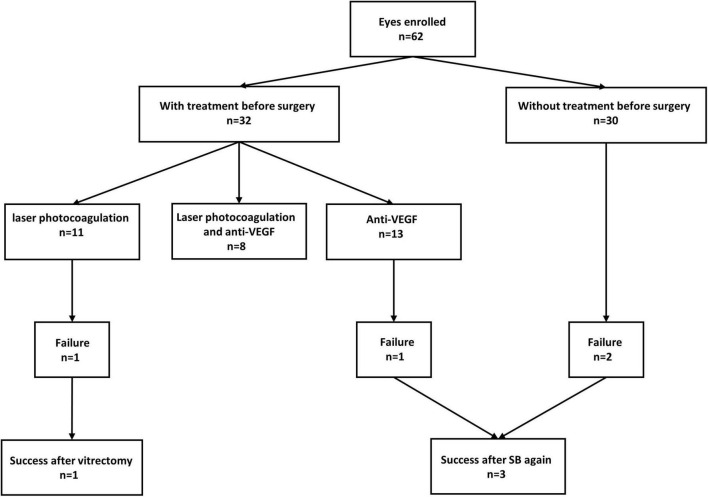
Flowchart of enrolled eyes with and without treatment before surgery and the outcome with different treatments. VEGF, vascular endothelial growth factor; SB, scleral buckling.

Traction retinal detachment spared the macula in all eyes, among which eight eyes (12.9%) had plus disease and four (6.5%) eyes had vitreous hemorrhage before surgery. Regarding previous treatments, 11 eyes (17.7%) had previously undergone laser photocoagulation therapy, 13 eyes (21.0%) only had anti-VEGF therapy, and 8 eyes (12.9%) had both anti-VEGF therapy and laser photocoagulation before surgery, as outlined in Figure 1. Thirty eyes (48.4%) had not received any treatment before surgery. For intraoperative combined treatment, nine eyes (14.5%) had combined cryotherapy, six eyes (9.7%) had combined anti-VEGF therapy, and three eyes (4.8%) had combined laser photocoagulation.

**FIGURE 2:**
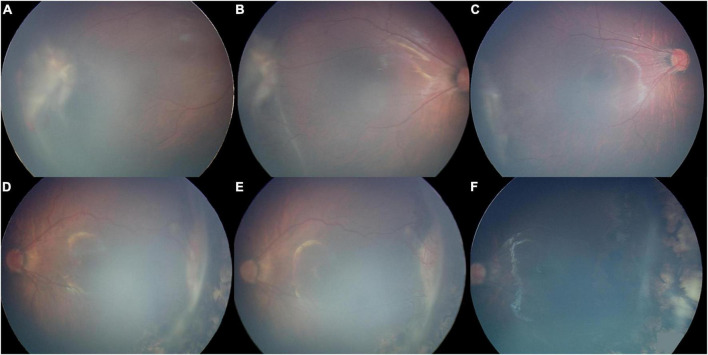
Fundus photography images from two patients in our series were obtained before and after surgery with stage 4A retinopathy of prematurity. Panels **A–C** belong to one patient. Panels **D–F** belong to another patient. **(A,D)** Fundus photograph obtained 1 week before scleral buckling showing extraretinal fibrovascular proliferation and tractional retinal detachment of the temporal retina. **(B,E)** Fundus photography obtained 1 week after scleral buckling, showing that the range of retinal detachment had no progression post-operatively. **(C,F)** Fundus photograph obtained about 6 months after scleral buckling, showing successful retinal reattachment.

The overall anatomic success rate, evaluated as the percentage of retinal attachment on ophthalmic evaluation, was 93.5% (58/62) after the first procedure and 100% after the second procedures. Figure 2 shows examples of fundus photography before and after SB in two patients. Among the patients for whom retinal attachment failed after the first procedure, three eyes showed successful retinal attachment after SB again, and one eye showed successful attachment after vitrectomy. Regarding the complications after the surgery, two eyes developed mild cataracts during the follow-up time, but lensectomy surgery was not needed. None of the operated eyes showed signs of glaucoma or other complications. Moreover, the average SE refraction value for patients was −3.00 ± 2.51 D (range −7.60 D to +2.75 D) 1 year after surgery.

## Discussion

Scleral buckling and LSV have been confirmed to be effective for stage 4A ROP. Although LSV has attracted much attention in the past few years, the inherent disadvantages, such as potentially iatrogenic retinal breaks, vitreous hemorrhage, and later severe proliferation after surgery, have caused wide concern. Compared with LSV, SB is less invasive and induces fewer surgical complications. However, the effectiveness of SB for stage 4A ROP has not been extensively studied.

Prior to 2003, the reattachment rates with SB were 66–75% (16–18). The initial retinal reattachment rate in our study was 93.5%. Futamura et al. (19) also reported a successful reattachment rate of 93.1% in a group of 29 eyes with stage 4A ROP after SB in 2014, which is comparable to our results. This finding suggests that the improved reattachment rates may be attributed to improvements in surgical equipment and diagnostic criteria over time. First, the considerable difference in the outcome seems to be related to the severity of the disease. Before 2003, studies followed the Cryotherapy for Retinopathy of Prematurity (CRYO-ROP) guidelines and always intervened at the threshold ROP. However, the Early Treatment for Retinopathy of Prematurity study (ETROP), published in 2003 (20), suggested that early treatment of high-risk prethreshold ROP, such as Type I ROP, can greatly improve the chance of long-term favorable structural outcomes, establishing new guidelines for the treatment of ROP. Various studies have confirmed the importance and effect of the adoption of the revised indications (21, 22). We believe that the final prognosis of disease is associated with the baseline disease condition at the time of treatment. Early treatment of prethreshold ROP can reduce the development of retinal detachment. So early treatment can reduce the severity of the disease when it comes to stage 4A ROP, such as the smaller range of retinal detachment. Therefore, this may explain the higher retinal reattachment rate in our study. Second, the average birth weight in previous studies ranged from 718 to 836 g, much less than that in our study, indicating more immature retinal nerve and vascular development and greater disease severity. Third, previous studies only combined laser photocoagulation therapy before surgery. In our study, most infants were also treated with anti-VEGF therapy, which can significantly improve the prognosis.

Several large series utilizing LSV for stage 4A ROP with adequate follow-up have reported favorable anatomical and functional success rates. In general, the successful reattachment rate of LSV for stage 4A ROP in previous studies ranged from 77 to 97% (10, 23–25). However, the complications caused by LSV surgery are a matter of great importance during the course of treatment. Bhende et al. (26) reported that the reattachment rate was 82% (23/29) in eyes with stage 4A ROP treated with LSV as the initial surgery. While there was significant intraoperative bleeding in 8 (27.6%) eyes, only one eye had such severe bleeding that completion of the surgery was hampered. Iatrogenic retinal breaks occurred in one eye, and corneal clouding occurred in one eye. Lakhanpal et al. (27) reported that 27 of 32 (84.4%) eyes were reattached after a single LSV, in which one eye had an intraoperative retinal tear and three eyes with vitreous haze postoperatively needed a secondary procedure. Nudleman et al. (28) found in a series that the reattachment rate after a single LSV surgery was 82.1% (230/280) for stage 4A ROP. However, the rate of lens opacity was 2.1%, and the rate of lensectomy following LSV was 12.5%. One study (29) also reported that two eyes (6.25%) had cataract formation, and one eye needed lensectomy. Chandra et al. (30) reported that 5 of 90 (5.56%) eyes with stage 4A ROP were noted to have glaucoma after successful LSV, in which one eye was subsequently controlled with ocular hypotensive medication whereas four eyes required filtering surgeries. There was also one study reporting that the incidence of glaucoma after LSV in stage 4A ROP stage was 6.9% (31). The incidence of cataracts in our series was 3.2% (2/62) and no lensectomy was required for lens opacity confined to the temporal side, which was significantly lower than LSV. In addition, no eyes developed glaucoma, vitreous hemorrhage or retinal breaks in our study.

In our study, 51.6% (32/62) of patients were also treated with anti-VEGF therapy or photocoagulation before the operation. The initial reattachment rate was 90.6% (29/32) in the combined treatment group. Futamura et al. (19) compared the results of SB surgery with or without intravitreal bevacizumab and photocoagulation for stage 4A ROP. They reported that the success rate was 92% in the combined group and 93% in the non-combined group, which is comparable to our cohort. However, they also found that the retinopathy was more severe in stage 4A ROP in the combined group because the proliferative tissue in this group was larger and the VEGF concentration in the aqueous humor was higher. This indicates that the combined surgery had some positive effect on the result of treatment. Ozsaygili et al. (32) also suggested that preoperative treatment improved the functional and anatomical success rates and reduced the incidence of some complications, such as vitreous hemorrhage and retinal tears. The success rate was 89.7% in the combined group in the first year after the initial procedure, but 50% in the non-combined group. Both anti-VEGF and photocoagulation can stabilize vascular activity and plus disease, reducing the activity of retinopathy and delaying the development of lesions. Performing SB or LSV to offset the tractional forces of the extraretinal proliferation in time when the lesions are quiet after combined treatment can yield encouraging outcomes.

Although SB can clearly lead to anatomic success, the high level of myopia induced by surgery and the requirement for a second procedure to divide or remove the buckle to allow normal ocular growth are concerns for many clinicians, limiting the widespread use of SB. In one series (33), myopia (−5 D to −15 D) was found in all ten eyes that underwent SB at stage 4A ROP with a mean follow-up of 35 months; in another series (34), the mean induced myopia was −7.75 DS (−6 D to −10.25 D) with a mean follow-up of 10.5 weeks. Although the myopia caused by SB in these studies was serious, the myopia caused by LSV is not optimistic. Holz et al. (35) reported that the average SE of nine eyes with a mean follow-up of 3.6 years treated with LSV for stage 4A ROP was −6.78 D. Another study (32) showed that the average SE of 82 LSV eyes with a mean follow-up of 22.6 months was −5.91 ± 3.47 D. However, the average SE refraction value for patients in our study was −3.00 ± 2.51 D (−7.60 D to +2.75 D) 1 year after surgery. One of the most important reasons why our data were less myopic than any previous study is that all of our procedures involved segmental SB instead of scleral encircling buckling. Thus, the postoperative refractive errors and eye distortions were minimal, and no one had to undergo a second procedure to remove the buckle.

This study has inherent limitations worthy of consideration. Our series was not randomized, controlled, or prospective in nature. In addition, the high variability in the duration of follow-up has the potential to bias the reported outcomes. The short-term anatomic success rate is often higher than long-term anatomic rate, and recurrence may be occurred up to 5–6 years. In our study, the mean time of the follow-up was 3.64 ± 2.53 years and only fourteen patients were followed for more than 5 years, accounting for 29.2% (14/48) of all patients. Future studies with longer follow-up periods are needed.

To our knowledge, this is the largest study in terms of sample size for stage 4A ROP treated with segmental SB to date. Compared with LSV, SB is certainly a relatively safe, low-cost and low-technology procedure. In conclusion, SB, especially segmental SB, is effective as an initial treatment for stage 4A ROP.

## Data availability statement

The raw data supporting the conclusions of this article will be made available by the authors, without undue reservation.

## Ethics statement

The studies involving human participants were reviewed and approved by the Ethics Committee and Institutional Review Board of Peking University People’s Hospital (2017PHB179-01). Written informed consent to participate in this study was provided by the participants’ legal guardian/next of kin.

## Author contributions

YC and JL conceived and designed the study. YZ, YY, HY, MZ, XL, and YC contributed important intellectual content to the study design and manuscript. YZ, YY, HY, and MZ coordinated data collection and contributed to the manuscript. YZ, YC, JL, and XL designed the analysis methods, analyzed the data, and contributed to manuscript writing. All authors contributed to the article and approved the submitted version.

## Abbreviations

SB: scleral buckling
ROP: retinopathy of prematurity
VEGF: vascular endothelial growth factor
LSV: lens-sparing vitrectomy
SE: spherical equivalent.
